# 3,4,5-Tri-*O*-Caffeoylquinic Acid Promoted Hair Pigmentation Through β-Catenin and Its Target Genes

**DOI:** 10.3389/fcell.2020.00175

**Published:** 2020-03-25

**Authors:** Meriem Bejaoui, Myra O. Villareal, Hiroko Isoda

**Affiliations:** ^1^School of Integrative and Global Majors (SIGMA), University of Tsukuba, Tsukuba, Japan; ^2^Faculty of Life and Environmental Sciences, University of Tsukuba, Tsukuba, Japan; ^3^Alliance for Research on the Mediterranean and North Africa (ARENA), University of Tsukuba, Tsukuba, Japan

**Keywords:** 3, 4, 5-tri-*O*-caffeoylquinic acid, β-catenin, melanocytes, anagen, melanogenesis enzymes, microphthalmia-associated transcription factor, melanin

## Abstract

The hair follicle undergoes a regular cycle composed of three phases: anagen, catagen, and telogen. The life of follicular melanocytes is totally linked to the hair cycle; and during anagen or the growth phase, the melanocytes are active and produce the melanin responsible of hair shaft pigmentation. Various signaling pathways regulate the hair growth cycle and, therefore, the pigmentation; we distinguish the Wnt/β-catenin signaling pathway as it plays a major role in the development, growth, and proliferation of the melanocytes and the activation of melanogenesis enzymes and the related transcription factor. In this study, 3,4,5-tri-*O*-caffeoylquinic acid (TCQA), a caffeoylquinic acid derivative, stimulated the pigmentation in C3H mouse hair follicle, in human melanocytes, and B16F10 melanoma cells. An enhancement in pigmentation associated genes was observed upon TCQA treatment *in vivo* and *in vitro*. Interestingly, the expression of β-catenin was remarkably upregulated in mouse treated skin and in pigment cell lines. Moreover, TCQA upregulated *CTNNB*1 expression after inhibition in human melanocytes. Taken together, this study suggests that TCQA triggered β-catenin activation to enhance the pigmentation during the anagen phase of the hair cycle.

## Introduction

In the adult hair follicle (HF), hair shaft pigmentation results from a coordinated and complicated cross talk between follicular melanocytes (FMs), matrix keratinocytes, and dermal papilla (DP) fibroblasts (Slominski and Paus, 1993; Slominski et al., 2005). Melanin-producing cells, the melanocytes, are originally derived from the neural crest cells, and their precursors, the melanoblasts, migrated to be localized in the HF and the epidermis (Nishimura et al., 1999). FMs and epidermal melanocytes share the same origin and melanin-producing function, but these melanocytes’ sub-population differ in many ways (Tobin et al., 2005). In the HF, melanogenically active melanocytes are located in the hair bulb, adjacent to the DP and the keratinocytes, whereas amelanotic melanocytes or melanocyte stem cells (MSCs) are found at the outer root sheath and in the bulge, a region known to contain HF stem cells (Tobin et al., 1999).

Mature HF undergoes a growth cycle consisting of phases of growth (anagen), regression (catagen), and rest (telogen) (Rosenquist and Martin, 1996). During the anagen phase, the FMs synthesize the pigment melanin that will be transferred in the melanosomes to the adjacent keratinocytes, also known as hair progenitors cells, that will be giving arise to the hair shafts (Nishimura et al., 2002; Lin and Fisher, 2007). The melanin synthesis is reduced starting in the catagen phase and be completely absent during the telogen phase, as the lower third of the HF, including the bulb region where the FMs are located, dies through apoptosis (Seiberg et al., 1995; Slominski, 2004; Nishimura, 2011). Therefore, the life of FMs is totally linked with the hair growth cycle, as they disappear when the HFs regress and reappear during the growing phase of the HFs (Steingrímsson et al., 2005; Nishimura, 2011). Melanin biosynthesis in the HF and the epidermis occurs through an oxidation process starting with L-tyrosine in the melanosomes that contain the related enzymes tyrosinase (TYR), tyrosinase-related protein 1 (TYRP1), and dopachrome tautomerase (DCT) (Kobayashi et al., 1994; Lin and Fisher, 2007). The expression of TYR, TYRP1, and DCT is regulated by the microphthalmia-associated transcription factor (MITF) known to play an important role in the development and survival of the melanocytes (Shibahara et al., 2004). Melanin synthesis, also called the melanogenesis, and pigment transfer to bulbar keratinocytes are under the regulation of various signal transduction pathways intrinsic to the skin and HF and determined by the availability of melanin precursors (Slominski et al., 2005). The important transcription factors involved in melanin production are the tyrosine kinase receptor KIT, its ligand SCF, MITF, and MC1R often established as the main factor dictating the melanogenesis (Hou et al., 2000; D’Mello et al., 2016). Mitf is targeted at the transcriptional level by several transcriptional factors including PAX3, CREB, SOX9, SOX10, and LEF1 and at post-transcriptional level involving mitogen-activated protein kinase (MAPK) and Wnt/β-catenin/GSK3β pathway (Levy et al., 2006; Passeron et al., 2007; Ngeow et al., 2018).

Recently, the use of plant-based products that may affect the regulation of pigmentation at the molecular level to promote melanogenesis has been approved in the mainstream of medicine (Schmidt et al., 2007; Villareal et al., 2017). In this context, caffeoylquinic acid (CQA) is a phenylpropanoid compound in which its derivatives exhibit a variety of bioactivities such as antioxidant, antibacterial, anticancer, antihistaminic, and melanogenesis-regulating effects (Kimura et al., 1985; Yoshimoto et al., 2002; Matsui et al., 2004; Kurata et al., 2011; Miyamae et al., 2011; Li et al., 2014; Kim et al., 2015). Among CQA derivatives, 3,4,5-tri-*O*-caffeoylquinic acid or TCQA with an International Union of Pure and Applied Chemistry (IUPAC) name (3*R*,5*R*)-3,4,5-tris[[(*E*)-3-(3,4-dihydroxyphenyl)prop-2-enoyl]oxy]-1-hydroxycyclohexane-1-carboxylic is reported to have a stable albumin affinity and has been found to increase adenosine triphosphate (ATP) production in human neuroblastoma SH-SY5Y cell, to induce adult neurogenesis, and to improve deficit of learning and memory in an aging model of eight senescence acceleration-prone mice (Sasaki et al., 2012, 2019). Furthermore, in our previous study, we demonstrated that TCQA promoted hair regrowth in 8-week-old C3H male and human DP cell proliferation through the upregulation of β-catenin expression leading to the initiation and elongation of the anagen phase of the hair growth cycle (Bejaoui et al., 2019). Interestingly, the Wnt/β-catenin signaling pathway plays as well an important role in the regulation of pigmentation, and β-catenin regulates Mitf expression and is involved in melanocyte development and MSC proliferation and differentiation (Widlund et al., 2002; Schepsky et al., 2006).

In the present study, we evaluated the effect of TCQA on the promotion of hair pigmentation *in vivo* through the activation of β-catenin and its target genes. Additionally, we have performed a global gene expression profiling using DNA microarrays to elucidate the pigmentation-promoting effects of TCQA. Furthermore, β-catenin expression along with Mitf and the melanogenesis enzymes was evaluated in human melanocytes (HMs) and in B16F10 murine melanoma cells.

## Materials and Methods

### 3,4,5-Tri-*O*-Caffeoylquinic Acid Preparation

Synthesized TCQA was used for the *in vivo* and *in vitro* experiments. TCQA was firstly dissolved in 70% ethanol and then diluted in purified water and cell culture medium for the *in vivo* and *in vitro* experiments, respectively. Dr. Kozo Sato from Synthetic Organic Chemistry Laboratories, the FUJIFILM Corporation (Kanagawa, Japan), kindly provided us with TCQA with 97% purity.

### Animal Experiment

For hair pigmentation experiment, 8-week-old male C3H mice purchased from Charles River Laboratories, Japan Inc. (Kanagawa, Japan), were used. All the mice were allowed to acclimate for 7 days to the laboratory conditions under controlled settings of temperature (21–23°C) and light (light:dark 12:12) with access to food and water, at the Gene Research Center of the University of Tsukuba. Then, C3H mice were randomly divided into two groups (*n* = 5): TCQA-treated group and control group treated with Milli-Q water (vehicle). The hair pigmentation is tightly linked to the anagen phase of the hair cycle, and at this age (8 weeks old), the HFs of C3H mice are at the telogen phase, so in order to induce the transition from the telogen to anagen phase, the mice were first anesthetized using isoflurane (Wako Pure Chemical Industries, Tokyo, Japan), and then the dorsal part was shaved using a hair clipper. The vehicle or 1% TCQA was applied topically at the shaved area for 1 month, and during this period, the mice were observed daily. The mice were then scarified by cervical spine dislocation, the hair from the treated area at the dorsal part was plucked, and the treated skin was collected and kept in −80°C. All animal procedures were approved by the Animal Study Committee of the University of Tsukuba and were handled according to the guidelines for the Care and Use of Animals.

### Melanin Assay *in vivo*

The hair was plucked from the shaved area at mouse dorsal skin using small sterilized forceps. With the use of a high-precision balance, 1.5 mg of the hair was weighed in Eppendorf tubes and then immersed in 1 M of NaOH for 2–4 h at 80–85°C under slight agitation. The tubes were mixed by inverting every 30 min and then centrifuged at 12,000 rpm for 5 min at room temperature (RT). The amount of melanin was determined after measuring the absorbance at 470 nm. The total melanin content was evaluated using the standard curve for synthetic melanin (Le Pape et al., 2008). Moreover, the plucked hair was visualized to detect any change in the color, using a contrast microscope (Leica Microsystems, Wetzlar, Germany), and the intensity of the color was assessed using ImageJ processing program (National Institutes of Health, Bethesda, United States).

### DNA Microarray

Microarray hybridization probes were generated from isolated RNA extracted from the skin treated with vehicle or TCQA using ISOGEN solution (Nippon Gene, Tokyo, Japan) following the manufacturer’s instructions. Briefly, the extracted RNA was amplified and biotin labeled as aRNA. Then, the fragmented, biotin-labeled aRNA was hybridized to the Affymetrix mouse 430 PM Array strips containing probes for 45,141 mouse genes. Hybridized arrays were washed and stained in GeneAtlas Fluidics Station. The GeneChip (Mouse Genome 430 2.0 Array) was scanned using the Affymetrix GeneAtlas Imaging Station to obtain the mRNA expression data of genes from mouse genome. The generated data (twofold change, control vs. TCQA) were then analyzed using Transcriptome Analysis Console (TAC) Software (version 4.0.1) and database for annotation, visualization, and integrated discovery (DAVID) bioinformatics resources 6.8 (Huang et al., 2009; Zacariotti et al., 2010).

### Immunohistochemistry

Skin tissues were collected from mice’s treated area, divided into four parts, and then embedded in optimum cutting temperature (OCT) compound. Therefore, the sections were cut at a thickness of 10 μm and fixed in 4% paraformaldehyde (Sigma, St. Louis, United States). A washing was then conducted using different solutions of phosphate-buffered saline (PBS), 0.1% v/v Triton X-100/PBS (Sigma, St. Louis, United States), and 20 mM of glycine/PBS. After incubation with the blocking solution [0.2 g of bovine serum albumin (BSA), 400 μl of gelatin, 18.4 ml of PBS, 500 μl of normal donkey serum, and 500 μl of normal goat serum], the sections were incubated with rabbit anti-tyrosinase (Abcam, Rockford, United States) and mouse anti-CD34 (Abcam, Rockford, United States) overnight at 4°C in a wet chamber. The skin sections were washed with the previously described washing solutions and then immersed in a solution of Alexa 594-conjugated anti-rabbit (Abcam, Rockford, United States) and Alexa Fluor 488-conjugated anti-mouse (Abcam, Rockford, United States). After being dried, the sections were stained with Hoechst, mounted in antifade solution (*p*-phenylenediamine, PBS, and glycerol), and visualized under a confocal microscope Leica, TCS, SP8 (Leica Microsystems, Wetzlar, Germany).

### Cells and Cell Culture

Human epidermal melanocytes (HEMs) were purchased from Gibco Invitrogen cell culture. HEMs were isolated from moderately pigmented (MP) neonatal foreskin (HEMn-MP) (GIBCO, Cat. No. C-102-5C). They are primary cells that can be cultured up to three or four passages before going into senescence. HEMs were maintained in Medium 254 (Gibco, MA, United States) supplemented with HM growth supplement HMGS (Gibco, MA, United States). HMGS contains bovine pituitary extract, fetal bovine serum, bovine insulin, bovine transferrin, basic fibroblast growth factor, hydrocortisone, heparin, and phorbol 12-myristate 13-acetate (PMA). PMA is known to induce melanocyte growth and melanin synthesis. The cells were seeded in Medium 254 supplemented with HMGS containing PMA (HMGS); but during the treatment, the cells were treated with medium supplemented with HMGS that does not contain PMA (HMGS-2) to test the effect of the sample on promoting the pigmentation.

B16 murine melanoma cells (B16F10) were purchased from the Riken Cell Bank in Tsukuba, Japan, and maintained under sterile conditions as a monolayer culture in Roswell Park Memorial Institute (RPMI) 1640 medium (Thermo Fisher Scientific, United Kingdom) supplemented with 10% fetal bovine serum (Gibco, MA, United States).

The cells were kept under sterile conditions at 37°C in a 75 cm^2^ flask (BD Falcon, United Kingdom) in a humidified atmosphere of 5% CO_2_. Trypan blue exclusion was used to determine the cell viability.

### Melanin Content and Cell Viability Determination

The method elaborated by Hosoi et al. (1985) was used to determine the melanin content with some changes. HEM and B16F10 were seeded at a density of 5 × 10^5^ cells per 100 mm petri dish and incubated at 37°C in a 5% CO_2_ atmosphere. After overnight incubation, the growth medium Medium 254 (HMGS) or RPMI 1640, respectively, for HEM or B16F10 was replaced with a fresh one containing different concentrations of TCQA, α-MSH [melanocyte-stimulating hormones (200 nM)] used as a positive control and XAV939 a β-catenin inhibitor. For the treatment, Medium 254 (HMGS-2) PMA free was used. After 48, 72, and 96 h, the growth medium was removed, and the cells were harvested by trypsinization (Gibco, MA, United States). The pelleted cells were solubilized by 0.1% Triton X-100 and precipitated in 10% trichloroacetate. The melanin was dissolved in 1 ml of 8 N NaOH and incubated for 2 h at 80°C. The amount of melanin was determined after measuring the absorbance at 410 nm. The total melanin content was evaluated using the standard curve for synthetic melanin (Le Pape et al., 2008). The melanin content was expressed as melanin content/cell (% of control). The number of viable cells used for melanin assay was quantified using the ViaCount program of Guava PCA (GE Healthcare, Buckinghamshire, United Kingdom), following the manufacturer’s instructions.

### Western Blot

HEM and B16F10 were seeded at density of 5 × 10^5^ cells per 100 mm petri dish and then treated with 0, 10, and 25 μM of TCQA, and 200 nM of α-MSH. After 4, 8, 12, 24, and 48 h, total protein extraction was achieved using radioimmunoprecipitation assay (RIPA) buffer (Sigma, St. Louis, United States) and protease inhibitor following the manufacturer’s instructions.

The quantification was assessed using a 2-D Quant kit according to manufacturer’s instructions (GE Healthcare, Chicago, United States).

The protein sample (15 μg/well) was separated in 10% sodium dodecyl sulfate–polyacrylamide gel electrophoresis (SDS-PAGE) and transferred to polyvinylidene difluoride (PVDF) membrane (Millipore, NJ, United States). After being blocked, the membranes were incubated with primary antibodies against β-catenin 71–2700 (Thermo Fisher Scientific, MA, United States), Mitf (Abcam, Rockford, United States), tyrosinase (Abcam, Rockford, United States), and Tyrp1 sc-166857, Dct sc-74439, and GAPDH sc32233 (Santa Cruz Biotechnology, TX, United States). After overnight incubation at 4°C, the second antibody goat anti-rabbit IRDye 800 CW or IRDye 680 LT goat anti-mouse was added. Then the expression was detected using LI-COR Odyssey Infrared Imaging System (LI-COR, NE, United States).

### Quantitative Real-Time PCR Analysis

The skin sections from mice’s treated area with TCQA or Milli-Q water were crushed using a homogenizer, and then the total RNA was extracted using an ISOGEN solution (Nippon Gene, Tokyo, Japan) following the manufacturer’s instructions.

The cells were seeded, allowed to attach overnight, and then treated with 10 and 25 μM of TCQA for HEM and B16F10, respectively, and with 200 nM of α-MSH for both cell lines. After 1, 6, 12, 24, and 48 h of treatment, the total RNA was extracted using ISOGEN kit (Nippon Gene, Tokyo, Japan) by following the manufacturer’s instructions and quantified using a NanoDrop 2000 spectrophotometer (NanoDrop Technologies, MA, United States).

The cDNA was synthesized from the extracted RNAs using SuperScript III reverse transcription kit (Invitrogen, CA, United States) with a cycling protocol, as follows: 95°C for 10 min, 40 cycles of 95°C for 15 s, and 60°C for 1 min. The real-time PCR was performed using 7500 Fast Real-Time PCR Software 1.3.1 (Applied Biosystems, CA, United States) with TaqMan probes specific to *Ctnnb1* (Mm 00483039_m1), *CTNNB1* (Hs99999168_m1), *Tyr* (Mm00495817_m1), *TYR* (Hs00165976_m1), *Tyrp1* (Mm004 53201_m1), *TYRP1* (Hs00167051_m1), *Dct* (Mm01225584_m1), *DCT* (Hs01098278_m1), *Mitf* (Mm00434954_m1), and *MITF* (Hs01117294_m1) (Applied Biosystems, CA, United States). *Gapdh* (Mm99999915_g1) and *GAPDH* (Hs02786624_g1) (Applied Biosystems, CA, United States) were used as an endogenous control. The 2^–ΔΔ*Ct*^ method was applied to calculate the relative mRNA expression levels using *Gapdh* and *GAPDH*.

### Immunostaining

The cells were seeded at a density of 3 × 10^4^/well in Lab-Tek Chamber Slides (Sigma, St. Louis, United States) and then allowed to attach overnight at 37°C. HEMs were treated with 0 and 10 μM of TCQA for 0, 4, 8, and 12 h. The medium was then removed, and the cells were washed with 0.1% v/v Triton X-100/PBS (Sigma, St. Louis, United States). After 1 h incubation at RT with the blocking solution (1.5 g of BSA, 50 ml of PBS, and 100 μl of Triton X-100), the first antibody rabbit anti-β-catenin (Abcam, Rockford, United States) prepared in the blocking solution was added overnight at 4°C. Therefore, the cells were immersed in a solution of 1:10,000 dilution of Alexa 594-conjugated anti-rabbit (Abcam, Rockford, United States) and mounted with DAPI before visualization under a confocal microscope Leica, TCS, SP8 (Leica Microsystems, Wetzlar, Germany).

### Statistical Analysis

All the experiments were run in triplicate. All results were expressed as mean ± standard deviation (SD). Student’s *t*-test was performed when two groups were compared. *P-*value of ≤ 0.05 was considered significant. For microarray analyses, a twofold change in the gene expression between control- and TCQA-treated groups was considered significant using ANOVA (one-way between-subject ANOVA, unpaired).

## Results

### 3,4,5-Tri-*O*-Caffeoylquinic Acid Promoted Pigmentation in C3H Mouse Hair Shaft

**FIGURE 1:**
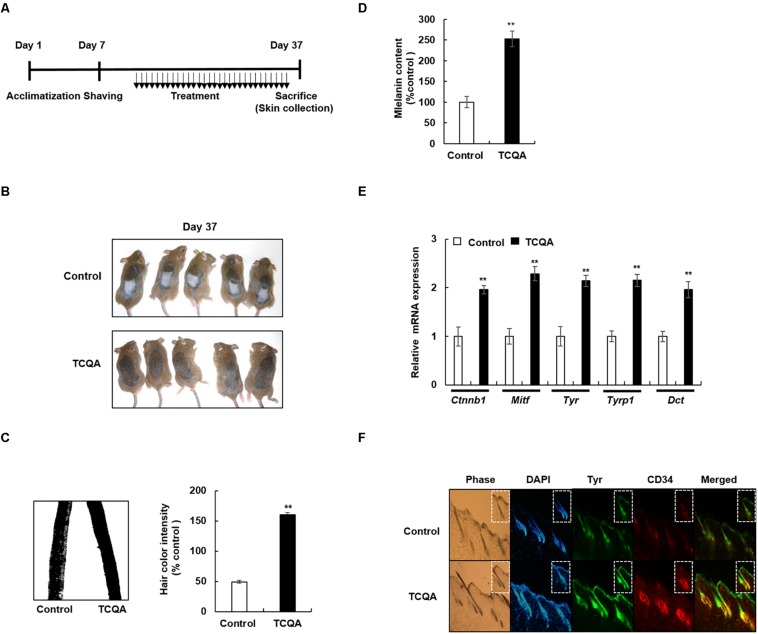
3,4,5-Tri-*O*-caffeoylquinic acid (TCQA) enhanced the pigmentation in 8-week-old C3H male mouse hair shaft. **(A)** Time table of the performed animal experiments. The dorsal part of the mice was shaved and treated daily for 30 days with topical application of 1% TCQA and the vehicle (Milli-Q water). Upon sacrifice, the treated skin and the hair shaft were collected. **(B)** Regeneration of a new darker hair in TCQA-treated group compared with the controls at day 37. **(C)** Hair plucked from the mice’s treated area and photographed under the microscope at day 37. Hair color quantification was measured by Image J. **(D)** Total melanin content was determined at the end of the treatment period from the plucked hair. **(E)** *Ctnnb1*, *Mitf*, *Tyr*, *Tyrp1*, and *Dct* mRNA relative expression was measured after treatment with TCQA at day 37. The mRNA level was quantified using TaqMan real-time PCR from RNA extracted from the treated area (TCQA or Milli-Q water) from mouse dorsal back. **(F)** Immunohistochemistry analysis of Tyr and CD34 in the hair follicle and the epidermis of the treated skin. The first panel is the phase, the second is DAPI to stain the nucleus, the third is for Tyr staining, the fourth is for CD34 staining, and the last panel is a combination of Tyr and CD34. **Statistically significant (*P* ≤ 0.01) difference between vehicle-treated mice and TCQA-treated mice.

To evaluate the effect of TCQA on hair pigmentation, the dorsal part of 8-week-old C3H mice was shaved and was then treated topically with TCQA and the vehicle for a month (Figure 1A). By the end of the treatment period, the mice were photographed, and results showed that the hair shaft from the dorsal part of TCQA-treated mice displayed a darker color than did that of the control (control vs. TCQA) (Figure 1B). To prove further this point, the hair from the treated area was plucked and photographed under the microscope, and the color intensity was measured. Results showed that the color intensity from TCQA-treated hair shaft is enhanced up to 3.26-fold compared with that of the control (Figure 1C). Moreover, melanin assay from the plucked hair was performed, and Figure 1D shows that the melanin content increased up to 175% after TCQA application. These results indicated that TCQA enhanced pigmentation of the hair shaft *in vivo*.

### 3,4,5-Tri-*O*-Caffeoylquinic Acid Affected the Expression of Pigmentation-Associated Genes in Mouse Collected Skin

To uncover the mechanism behind the enhancement of pigmentation by TCQA, microarray analysis was conducted. Tables 1, 2 illustrate a summary of the top regulated genes. Pigmentation-associated genes were upregulated with TCQA treatment (Table 1). The gene expression of *Mitf* was enhanced up to 2.17-fold compared with that of the control (Table 1). This upregulation was followed by an increase in the expression of *Tyr*, *Tyrp 1*, and *Dct* up to, respectively, 2. 34-, 2. 23-, and 2.28-fold. Moreover, β-catenin expression was stimulated, and this is the most likely cause for *Mitf* upregulation as it is a transcription factor stimulating Mitf activation (Table 1). The upregulation of other transcription factors including *Pax3*, *Stat3*, *Sox10*, and *Creb* was also observed. In our previous study, genes with a twofold change in expression (control vs. TCQA) were subjected to hierarchical clustering that generated five clusters. In the second and third clusters, the regulated genes were significant in ATP binding, Wnt signaling, cAMP signaling pathway, and neural cell differentiation and migration (Bejaoui et al., 2019). Microarray results showed as well a stimulation in the expression of *Cdh11* involved in melanogenesis promotion and *Agmo* playing a role in the reduction of reactive oxygen species (ROS) emission (Kim et al., 2014; Table 1).

**TABLE 1:** Top upregulated pigmentation-associated genes in TCQA-treated mice (vs. control) *.

**Gene symbol**	**Gene name**	**Biological function**	**Fold-change**	*P-*value**
*Cdh11*	Cadherin 11	Influence the melanin biosynthesis *via* Wnt pathway	5.37	0.005
*Pax3*	Paired box 3	DCT regulation; neural crest migration; MITF activation	3.38	0.026
*Ctnnb1*	Catenin (cadherin associated protein), beta 1	Melanocyte stem cells differentiation; MITF activation; pigmentation	3.33	0.043
*Fzd2*	Frizzled class receptor 2	Canonical Wnt signaling pathway activation	2.89	0.035
*Creb*	cAMP-response element binding protein	Transcription of MITF	2.67	0.031
*Stat3*	Signal transducer activator of transcription 3	PAX3 activation; melanocyte viability	2.34	0.004
*Tyr*	Tyrosinase	Melanin biosynthetic process; melanocytes proliferation	2.34	0.002
*Dct*	Dopachrome tautomerase	Melanin biosynthetic process; melanocyte development	2.28	
*Tyrp1*	Tyrosinase-related protein 1	Melanin biosynthetic process; Melanosome membrane	2.23	0.085
*Agmo*	Alkylglycerol monooxygenase	Reduction of ROS emission	2.22	0.021
*Mitf*	Microphthalmia-associated transcription factor	Pigmentation; positive regulation of transcription; melanocyte differentiation	2.17	0.007
*Mc1r*	Melanocortin 1 receptor	Melanin biosynthetic process; pigmentation	2.06	0.002
*Sox10*	SRY (sex determining region Y)-box 10	Melanocyte differentiation in the hair follicle; pigmentation	2.06	0.003
*Kitl*	Kit ligand	Neural crest cell migration; melanocytes proliferation	2.03	0.015

TCQA, 3,4,5-tri-O-caffeoylquinic acid; DCT, dopachrome tautomerase; MITF, microphthalmia-associated transcription factor; ROS, reactive oxygen species. *Gene functions were obtained from Mouse Genome Informatics (MGI). **ANOVA was performed to assess the level of significance between groups. The gene expression was considered significant when fold change was ≥ 2-fold (control vs. TCQA).

**TABLE 2:** Top downregulated genes in TCQA-treated mice (vs. control) *.

**Gene symbol**	**Gene name**	**Biological function**	**Fold-change**	*P-*value**
*Jun*	Jun proto-oncogene	JNK pathway; downregulation of melanogenesis	−4.62	0.049
*Hdac2*	Histone deacetylase 2	Negative regulation of canonical Wnt signaling	−4.49	0.044
*Cox-2*	Cyclooxygenase-2	Inducible nitric oxide synthase; implication in melanoma	−4.13	0.016
*ATG6*	Autophagy related 6	Autophagy; melanin degradation	−3.96	0.023
*Rps6*	Ribosomal protein S6	TOR hyperactivation; melanin degradation	−3.44	0.025
*Jkamp*	JNK1/MAPK8-associated membrane protein	Activation by UV radiation; MAPK activation	−2.96	0.023
*Lamtor4*	Late endosomal/lysosomal adaptor, MAPK and MTOR activator 4	Positive regulation of TOR signaling	−2.72	0.010
*Mmp2*	Matrix metallopeptidase 2	Involvement in skin damage	−2.72	0.044
*Sirt1*	Sirtuin 1	Negative regulation of NF-κB signaling	−2.63	0.046
*Akt*	Thymoma viral proto-oncogene 1	Inhibition of melanogenesis	−2.22	0.030
*Wipi1*	WD repeat domain, phosphoinositide interacting 1	Autophagy; modulating melanogenesis	−2.22	0.019
*Lamtor5*	Late endosomal/lysosomal adaptor, MAPK and MTOR activator 5	Activation of mTOR pathway promoting tumorigenesis; blocking melanin synthesis	−2.18	0.020
*Mdm4*	Transformed mouse 3T3 cell double minute 4	Negative regulation of apoptotic process	−2.08	0.043
*Bcl-2*	B cell leukemia/lymphoma 2	Cell aging; proapoptotic and antiapoptotic regulators of apoptosis	−2.06	0.021

TCQA, 3,4,5-tri-O-caffeoylquinic acid; MAPK, mitogen-activated protein kinase. *Genes functions were obtained from Mouse Genome Informatics (MGI). **ANOVA was performed to assess the level of significance between groups. The gene expression was considered significant when fold change was ≥2-fold (control vs. TCQA).

On the other hand, genes significant for Wnt repression, melanin degradation, and melanogenesis inhibition were downregulated after TCQA treatment including *Jun*, *Hdac2*, *Rps6*, and *Akt*. In Table 2, we observed a downregulation in MAPK and mTOR pathway-associated genes involved in the inhibition of melanin biosynthesis and in genes relevant for autophagy and apoptosis.

Furthermore, a validation for the microarray results was carried out. Results showed that the gene expression of *Tyr*, *Tyrp1*, and *Dct* was enhanced up to twofold compared with that of the control. *Ctnnb1* and *Mitf* gene expression was upregulated up to, respectively, 2- and 2.2-fold, in TCQA-treated mouse skin (Figure 1E).

### 3,4,5-Tri-*O*-Caffeoylquinic Acid Upregulated Tyrosinase and CD34 Protein Expression in Mouse Treated Skin

Tyrosinase (Tyr) is considered as a marker of active melanocytes capable of producing the melanin during the anagen phase of the hair cycle (Kobayashi et al., 1994; Lin and Fisher, 2007). Microarray analysis showed an upregulation in pigmentation-associated genes including *Tyr.* To further investigate the effect of TCQA on melanocyte activation, Tyr protein expression was checked in mouse treated skin. Results revealed that in TCQA-treated mouse skin, Tyr expression was observed to be enhanced in the epidermis and in the bulb area of the HFs, suggesting the activation of the bulbar melanocytes and therefore the enhancement of pigmentation in mouse hair shaft (Figure 1F).

On the other hand, the expression of CD34 expressed in the bulge region of the HF during the anagen phase and considered to be a marker of MSCs that have the ability to differentiate into active melanocytes (Trempus et al., 2007; Joshi et al., 2019) was determined as well. CD34 expression was enhanced upon TCQA treatment in the outer root sheath and the bulge area of the HF where the MSCs are located (Figure 1F). This result showed that the HFs from TCQA-treated mice are in the anagen phase of the hair cycle and that TCQA induced the differentiation of MSCs. Altogether, TCQA induced the anagen phase of the hair growth cycle in the HF of C3H mice *via* the activation of β-catenin, leading to the upregulation of pigmentation-associated genes and therefore to the enhancement of the melanin biosynthesis.

### Effect of 3,4,5-Tri-*O*-Caffeoylquinic Acid on Melanin Biosynthesis in Human Epidermal Melanocytes and in B16F10 Murine Melanoma Cells

**FIGURE 2:**
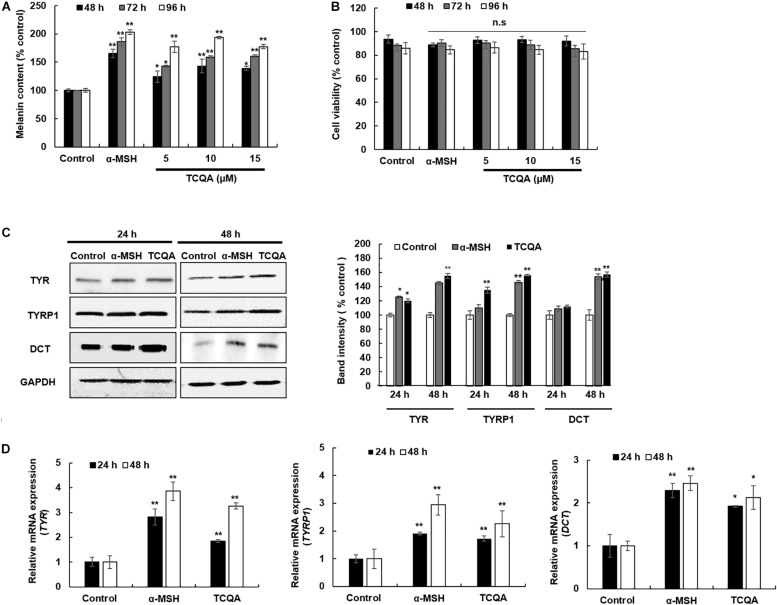
3,4,5-Tri-*O*-caffeoylquinic acid (TCQA) stimulated the melanogenesis in human epidermal melanocyte (HEM). **(A)** Melanin content determination after 48, 72, and 96 h of treatment with various concentrations of TCQA and 200 nM of α-MSH used as positive control. **(B)** Cell viability determination. **(C)** Determination of the protein expression of the melanogenesis enzymes – TYR, TYRP1, and DCT – after 24 and 48 h of treatment with 0 and 10 μM of TCQA and 200 nM of α-MSH. The band intensities were done by comparing GAPDH using LI-COR system. **(D)** Gene expression of *TYR*, *TYRP1*, and *DCT* after 24 and 48 h with 0 and 10 μM of TCQA and 200 nM of α-MSH. The mRNA level was quantified using TaqMan real-time PCR. Results represent the mean ± SD of three independent experiments. *Statistically significant (*P* ≤ 0.05) difference between control and treated cells. **Statistically significant (*P* ≤ 0.01) difference between control and treated cells.

**FIGURE 3:**
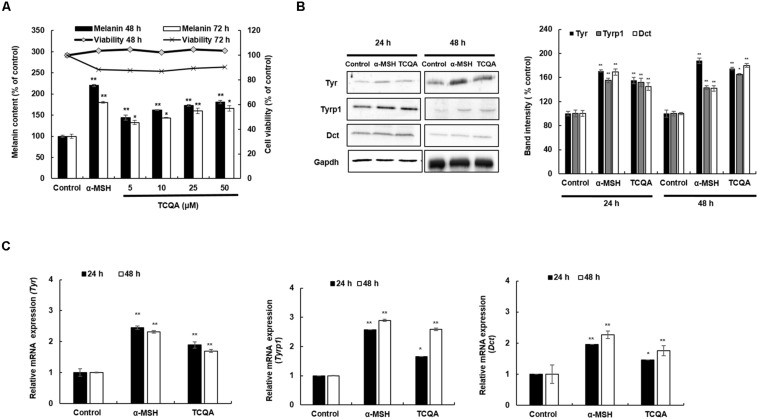
3,4,5-Tri-*O*-caffeoylquinic acid (TCQA) enhanced the pigmentation in B16F10 murine melanoma cells. **(A)** Melanin content and cell viability determination after 48 and 72 h of treatment with various concentrations of TCQA and 200 nM of α-MSH used as positive control. **(B)** Determination of the protein expression of the melanogenesis enzymes – Tyr, Tyrp1, and Dct – after 24 and 48 h of treatment with 0 and 25 μM of TCQA and 200 nM of α-MSH. The band intensities were done by comparing GAPDH using LI-COR system. **(C)** Gene expression of *Tyr*, *Tyrp1*, and *Dct* after 24 and 48 h with 0 and 25 μM of TCQA and 200 nM of α-MSH. The mRNA level was quantified using TaqMan real-time PCR. Results represent the mean ± SD of three independent experiments. *Statistically significant (*P* ≤ 0.05) difference between control and treated cells. **Statistically significant (*P* ≤ 0.01) difference between control and treated cells.

A melanin assay was performed to evaluate the effect of TCQA *in vitro* using mouse and human cell lines, which are widely used in pigment cell studies. HEM and B16F10 were treated with various concentrations of TCQA for 48, 72, and 96 h. Results showed that 10 μM of TCQA, compared with the control, increased the melanin content in HEM by 30, 50, and 93% after 48, 72, and 96 h, respectively (Figure 2A). Moreover, 25 μM of TCQA, compared with the untreated cells, significantly enhanced the melanin content in B16F10 by 70 and 62%, respectively, after 48 and 72 h as shown in Figure 3A. The effect of TCQA on the melanin biosynthesis was observed without decreasing the cell viability of both cell lines (Figures 2B, 3A). These results indicated that TCQA stimulated the melanogenesis in pigment cell lines.

### 3,4,5-Tri-*O*-Caffeoylquinic Acid Enhanced the Expression of the Melanogenesis Enzymes in Human Epidermal Melanocyte and B16F10 Cells

In mouse treated skin, the gene expression of *Tyr*, *Tyrp1*, and *Dct* was enhanced (Table 1 and Figures 1E,F). So a further investigation of the expression of the melanogenesis enzymes was carried out in HEM and B16F10. Results showed that the protein expression of TYR, TYRP1, and DCT was significantly enhanced in HEM after 48 h of treatment with 10 μM of TCQA (Figure 2C). *TYR*, *TYRP1*, and *DCT* gene expression was upregulated after 24 and 48 h of treatment with TCQA in HEM (Figure 2D). Furthermore, these results were as well validated in B16F10, in which the gene and the protein expression of these enzymes were significantly stimulated after 24 and 48 h of treatment with 25 μM of TCQA (Figures 3B,C). These results suggest that TCQA regulated the melanin synthesis by stimulating the protein and the gene expression of the melanogenesis enzymes.

### Microphthalmia-Associated Transcription Factor Expression Was Upregulated After Treatment With 3,4,5-Tri-*O*-Caffeoylquinic Acid in Human Epidermal Melanocytes and B16F10 Cells

**FIGURE 4:**
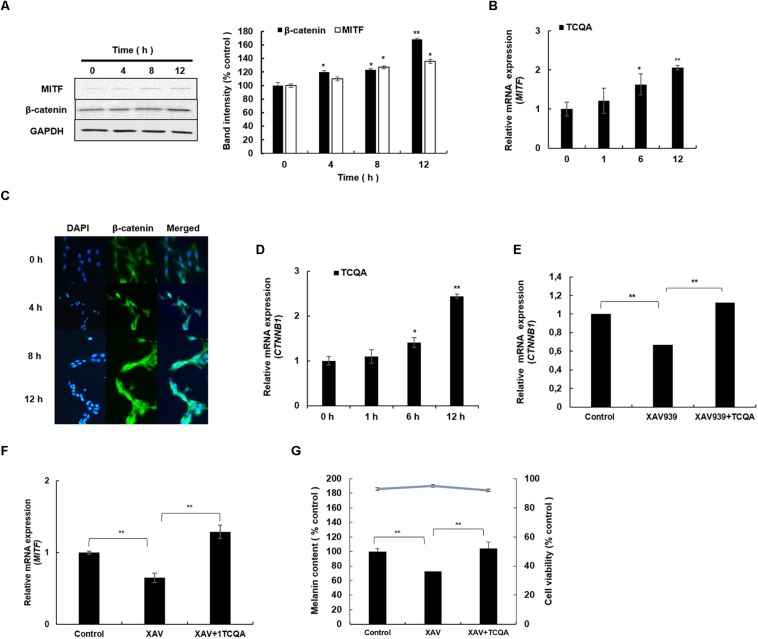
3,4,5-Tri-*O*-caffeoylquinic acid (TCQA) stimulated β-catenin and microphthalmia-associated transcription factor (MITF) expression in human epidermal melanocytes (HEMs). **(A)** Protein expression of MITF and β-catenin after 0, 4, 8, and 12 h of treatment with 10 μM of TCQA. **(B)** Gene expression of *MITF* after 0, 1, 6, and 12 h of treatment with 10 μM of TCQA. **(C)** Immunocytochemistry of β-catenin expression after 0, 4, 8, and 12 h of treatment with 10 μM of TCQA. Scale bar = 25 μm; magnification at 20×. **(D)** Gene expression of *CTNNB1* after 0, 1, 6, and 12 h of treatment with 10 μM of TCQA. **(E)** Gene expression *CTNNB1* after treatment with 10 μM of XAV939 and co-treatment of 10 μM of XAV939 and 10 μM of TCQA for 24 h. **(F)** *MITF* gene expression after treatment with 10 μM of XAV939 and co-treatment of 10 μM of XAV939 and 10 μM of TCQA for 24 h. **(G)** Melanin assay after 48 h of treatment with 10 μM of XAV939 and co-treatment of 10 μM of XAV939 and 10 μM of TCQA for 48 h. The band intensities were done by comparing GAPDH using LI-COR system; and the mRNA level was quantified using TaqMan real-time PCR. Results represent the mean ± SD of three independent experiments. *Statistically significant (*P* ≤ 0.05) difference between control and treated cells. **Statistically significant (*P* ≤ 0.01) difference between control and treated cells.

**FIGURE 5:**
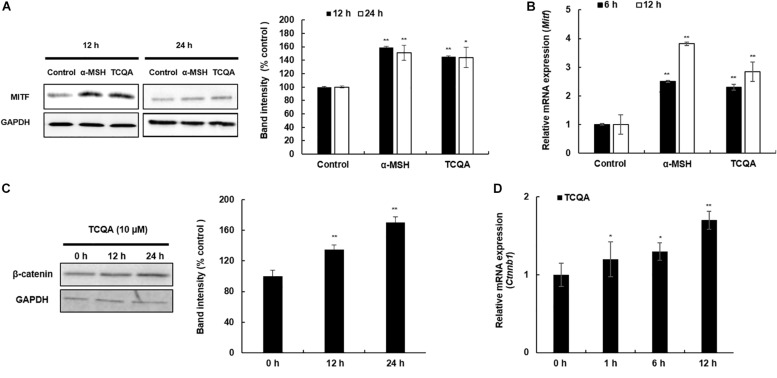
3,4,5-Tri-*O*-caffeoylquinic acid (TCQA) enhanced microphthalmia-associated transcription factor (MITF) and β-catenin expression in B16F10 murine melanoma cells. **(A)** Protein expression of MITF after 12 and 24 h of treatment with 0 and 25 μM of TCQA and 200 nM of α-MSH. GAPDH 24 h from Figure 3 band is reused in this figure. **(B)** Gene expression of *MITF* after 6 and 12 h of treatment with 0 and 25 μM of TCQA and 200 nM of α-MSH. **(C)** Protein expression of β-catenin expression after 0, 12, and 24 h of treatment with 25 μM of TCQA. **(D)** Gene expression of *Ctnnb1* after 0, 12, and 24 h of treatment with 25 μM of TCQA. The band intensities were done by comparing GAPDH using LI-COR system; and the mRNA level was quantified using TaqMan real-time PCR. Results represent the mean ± SD of three independent experiments. *Statistically significant (*P* ≤ 0.05) difference between control and treated cells. **Statistically significant (*P* ≤ 0.01) difference between control and treated cells.

The protein and the gene expression of MITF, known as the melanogenesis master regulator, was checked in HEM and B16F10. Figure 4A displays the result of MITF protein expression, which was upregulated time dependently after treatment with 10 μM of TCQA in HEM. The gene expression was enhanced as well time dependently after TCQA treatment in HEM (Figure 4B). In B16F10, 25 μM of TCQA upregulated Mitf protein expression after 12 and 24 h and the gene expression after 6, 12, and 24 h (Figures 5A,B).

### 3,4,5-Tri-*O*-Caffeoylquinic Acid Stimulated β-Catenin Expression in Human Epidermal Melanocyte and B16F10 Cells

β-Catenin regulates Mitf expression and influences skin and hair shaft pigmentation. Microarray analysis showed an upregulation in β-catenin gene expression (*Ctnnb1*) in mouse skin (Table 1 and Figure 1E). Moreover, our previous results showed that TCQA stimulated hair growth *in vivo* and *in vitro* through the activation of β-catenin (Bejaoui et al., 2019). Further investigation of the expression of β-catenin in HEM and B16F10 was carried out after treatment with TCQA. Results showed that the protein expression was enhanced time dependently with 10 μM of TCQA in HEM (Figure 4A). These results were supported by immunostaining analysis showing the accumulation and translocation of β-catenin to the nucleus of the melanocytes to activate target genes (Figure 4C). Figure 4D shows the upregulation of *CTNNB1* up to 2.6-fold after 12 h of treatment with 10 μM of TCQA. Additionally, the protein and the gene expression of β-catenin were upregulated time dependently in B16F10 (Figures 5C,D). These results suggest that TCQA enhanced pigmentation through the upregulation of β-catenin and its target Mitf leading to the activation of the melanogenesis enzymes and the stimulation of melanin biosynthesis.

### 3,4,5-Tri-*O*-Caffeoylquinic Acid Upregulated β-Catenin and Mitf Expression and the Melanin Content in Human Epidermal Melanocyte After Inhibition With XAV939

In this study, we suggested that the observed pigmentation promotion effect of TCQA was due to the activation of β-catenin triggering Mitf transcription and therefore promoting the melanin content. For this purpose, HEMs were treated with XAV939, an inhibitor of β-catenin activation, causing its phosphorylation and non-translocation into the nucleus (Kulak et al., 2015). HEMs were treated with only 10 μM of XAV939 and co-treated with TCQA and XAV939 for 24 h. Results revealed that 10 μM of XAV939 decreased significantly *CTNNB1* and *MITF* gene expression compared with the control, confirming the inhibitory effect of XAV939 on Wnt/β-catenin signaling leading to the inhibition of MITF transcription (Figures 4E,F). For the cells co-treated with 10 μM of XAV939 and 10 μM of TCQA, results showed that treatment with TCQA significantly upregulated *CTNNB1* and *MITF* expression, suppressing XAV939 inhibition (Figures 4E,F). To establish further the link between the activation of β-catenin by TCQA and the enhancement of pigmentation, HEMs were treated with XAV939 only and co-treated with XAV939 and TCQA for 48 h, and melanin content was determined. Results revealed a decrease of melanin content upon XAV939 treatment, and a significant increase was observed after the co-treatment with TCQA (Figure 4G). These results further proved that the activation of β-catenin by TCQA in HEM promoted melanin biosynthesis.

## Discussion

In the adult HF, the activity of FMs located in the hair bulb is under a cyclical control, and the melanin biosynthesis or the melanogenesis is tightly coupled to the hair growth cycle (Slominski, 2004; Tobin et al., 2005).

In our previous study, we demonstrated the activation of Wnt/β-catenin pathway with a polyphenolic compound TCQA to promote hair growth *in vivo* and in DP cells (Bejaoui et al., 2019). In this study, we showed that TCQA stimulated hair pigmentation *via* the upregulation of β-catenin and its target genes leading to the stimulation of the melanogenesis in 8-week-old C3H mice, in HMs, and in B16F10 murine melanoma cells.

TCQA enhanced the pigmentation and the melanin content in the hair shaft of C3H mice compared with the control (Figure 1). Mouse follicular pigmentation is under a complex genetic control involving more than 150 alleles, which allows the control of melanin synthesis at all levels from the developmental step of the melanocytes (differentiation from neural crest cells and the migration to the HF) until the pigmentation of the hair shaft (Slominski et al., 2005). Here, microarray analysis showed an upregulation of pigmentation-associated genes (Table 1). The biosynthesis of melanin or melanogenesis is a result of a series of transformations and reactions engaging L-tyrosine and mediated by tyrosinase (TYR), tyrosinase-related protein 1 (TYRP1), and DCT (Riley, 1997; Fuller et al., 2000). The gene expression of the melanogenesis enzymes *Tyr*, *Tyrp1*, and *Dct* was significantly upregulated upon treatment with TCQA in mouse collected skin (Table 1 and Figure 1E). Additionally, these enzymes are considered as markers of melanocytes and often used in histological analysis, allowing an accurate classification of the type and the stage of melanocytes, and the location in the HF (Murtas et al., 2019). FMs are classified into MSCs located in the HF bulge and express only DCT, differentiating and proliferating outer root sheath melanocytes expressing DCT and weakly TYRP1, and bulbar melanocytes expressing the three enzymes and actively producing the pigment melanin (Botchkareva et al., 2003; Botchkarev and Gilchrest, 2017). Furthermore, MSCs can also be marked by CD34 expressed during the anagen phase of the hair cycle and defining the stem cell subpopulations with distinct regenerative properties (Trempus et al., 2007; Joshi et al., 2019). In this study, histological analyses were performed to label the FMs and MSCs using tyrosinase (Tyr) and CD34 in mouse treated skin, and results showed that TCQA upregulated Tyr expression in the epidermis and along the HF especially in the bulb region, and this correlates with the activation of FMs and the production of melanin during the anagen phase of the hair cycle (Figure 1F). In addition, TCQA-treated mice displayed a strong expression of CD34 in the bulge region and the outer and inner root sheath of the HF, and this result showed that TCQA appears to activate MSCs to differentiate into melanin-producing cells (Figure 1F). The upregulation of the three melanogenesis enzymes can be attributed to the activation of the *Mitf* in mouse skin upon TCQA treatment as illustrated in Table 1 and Figure 1E. MITF plays a crucial role in the regulation of melanocyte development, function, and survival; and mice carrying null alleles of *Mitf* gene showed a loss of melanocytes derived from the neural crest cells (Moore, 1995; Goding, 2000; Villareal et al., 2017). Mitf transcription and therefore melanin production are initiated and regulated by a number of signaling systems and transcription factors. The role of MCR1, in which its activation increases cAMP synthesis, inducing a switch from the production of pheomelanin (red pigment) to the production of eumelanin (black pigment), and the SCF-KIT pathway involved in melanocyte pigmentation and development, are established in the activation of MITF (Flaherty et al., 2012; Bonaventure et al., 2013; D’Mello et al., 2016). TCQA enhanced the expression of transcription factors that regulate MITF expression including *Sox10*, *Creb*, *Mc1r*, and *Kitl* (Table 1). Although MITF is considered to be a major regulator of the melanogenesis, it has been reported that alone it cannot completely activate TYR gene expression (Gaggioli et al., 2003; Kawakami and Fisher, 2017). Recently, the role of STAT3 in stimulating the transcription of TYR by α-MSH is established (Shin et al., 2019). Moreover, STAT3 transactivated PAX3, which plays an important role in neural crest cells and melanocyte development, melanin level, and Mitf transcription, through a communication with FGF2 (Pingault et al., 2010; Dong et al., 2012). In this study, the gene expression of *Stat3* and *Pax3* was upregulated in TCQA-treated mouse skin, and in our previous results, we have reported that TCQA upregulated *Fgf2* gene expression in C3H mouse skin (Table 1; Bejaoui et al., 2019). FGF2 is required as well in the stimulation of hair growth cycle, and the expression may be controlled by Wnt/β-catenin signaling, as the absence of β-catenin alters FGF signaling genes (Tsai et al., 2014; Takabayashi et al., 2016). It has been revealed also that Wnt/β-catenin pathway is involved in hair pigmentation, as it regulates the hair cycle. In fact, β-catenin regulates positively *Mitf* expression at the transcriptional level, and the development, survival, and differentiation of melanocyte during formation and maturation (Dorsky et al., 2000; Jouneau et al., 2000; Yamada et al., 2013). Moreover, Wnt/β-catenin and KIT signaling sequentially regulate MSC differentiation and pigmentation (Yamada et al., 2013). β-Catenin gene expression and its receptor *Fzd2* were enhanced after TCQA treatment, and this may explain the activation of *Mitf* and therefore the melanogenesis enzymes, and the enhanced of melanin content in the hair shaft of the treated mice (Table 1 and Figure 1E).

During melanin synthesis, ROS are produced *via* the oxidation of L-tyrosine, and this leads to the depletion of MSCs pool, melanocyte damage, and skin and hair shaft aging (Nishimura, 2011; Tobin, 2011; Stout and Birch-Machin, 2019). Here, after treatment with TCQA, which is a polyphenolic compound with antioxidant properties, we found an enhancement in the gene expression of *Agmo* implicated in the oxidation-reduction process (Table 1). We have reported as well an upregulation of *CDH11* in Table 1, a gene expressed specifically in fibroblasts and keratinocytes and may influence the melanin biosynthesis *via* the canonical Wnt pathway (Kim et al., 2014).

Table 2 summarizes the downregulated genes upon treatment with TCQA; mainly, we observed a repression of genes involved in the inhibition of melanogenesis, autophagy, mTOR and AKT pathway, and cell aging. Melanogenesis is a process that is affected by autophagy, as it participates in melanosome degradation, and an impaired melanosome function allows rampant UVR penetration and melanin degradation. Moreover, the AKT/mTOR pathway is one of the well-studied pathways involved in autophagy, cell proliferation, and cell survival (Chen et al., 2014; Yang et al., 2018).

HEMs, melanin-producing cells, and B16 murine melanoma cells (B16F10) were used to further validate the effect of TCQA on pigmentation. Melanin content along with the gene and protein expression of TYR, TYRP1, and DCT was upregulated upon treatment (Figures 2, 3). This effect is attributed to the activation of MITF (gene and protein expression) by TCQA (Figures 4, 5). TCQA stimulated the pigmentation *via* the activation of β-catenin protein and genes expression in both cell lines (Figures 4, 5). Additionally, β-catenin was inhibited in HEM after treatment with XAV939, which is a small molecule that inhibits Tankyrase, known to activate canonical Wnt pathway, and this will lead to β-catenin phosphorylation and degradation (Kulak et al., 2015). The inhibitory effect of XAV939 on β-catenin on melanocytes cells has not yet been assessed. Here, upon XAV939 treatment, the gene expression of β-catenin and *MITF* was significantly decreased along with the melanin content in HEM, but a co-treatment with TCQA lifted and reduced the inhibition (Figures 4E–G).

Taken together, our findings suggest that TCQA triggered the activation of Wnt/β-catenin pathway leading to the upregulation of pigmentation-associated genes and the enhancement of melanin content in C3H mouse hair, as well as HMs and B16F10 murine melanoma cells. The downregulation of genes linked to autophagy and melanin degradation contributed to the further enhancement of the effect of TCQA on the stimulation of melanogenesis.

Furthermore, our previous study showed that the β-catenin-mediated hair growth induction effect of TCQA caused the initiation and elongation of the anagen phase of the hair cycle in C3H mice and the activation of human DP cells. In this context, the potential effect of TCQA on the activation of melanocytes through a communication with DP cells *via* Wnt/β-catenin will be looked at. Clinical studies would be, however, necessary to introduce TCQA as a potential candidate to initiate the hair growth cycle and to enhance melanogenesis.

## Data Availability Statement

The data has been deposited to NCBI, GEO database: https://www.ncbi.nlm.nih.gov/geo/query/acc.cgi?acc=GSE138644 (accession: GSE138644).

## Ethics Statement

The animal study was reviewed and approved by the Animal Study Committee of the University of Tsukuba, and were handled according to the guidelines for the Care and Use of Animals.

## Author Contributions

MB performed most of the experiments, analyzed the data, and wrote the manuscript. MV helped in the design of the experiments and data analysis. HI conceived and supervised the study and reviewed and approved the manuscript. All authors read and approved the final manuscript.

## Conflict of Interest

The authors declare that the research was conducted in the absence of any commercial or financial relationships that could be construed as a potential conflict of interest.
